# Peanut (*Arachis hypogaea*) Expressed Sequence Tag Project: Progress and Application

**DOI:** 10.1155/2012/373768

**Published:** 2012-06-17

**Authors:** Suping Feng, Xingjun Wang, Xinyou Zhang, Phat M. Dang, C. Corley Holbrook, Albert K. Culbreath, Yaoting Wu, Baozhu Guo

**Affiliations:** ^1^Department of Plant Pathology, University of Georgia, Tifton, GA, USA; ^2^College of Bioscience and Biotechnology, Qiongzhou University, Sanya, China; ^3^High-Tech Research Center, Shandong Academy of Agricultural Sciences, Jinan, China; ^4^Henan Academy of Agricultural Sciences, Zhengzhou, China; ^5^National Peanut Research Laboratory, USDA-ARS, Dawson, GA, USA; ^6^Crop Genetics and Breeding Research Unit, USDA-ARS, Tifton, GA, USA; ^7^Crop Protection and Management Research Unit, USDA-ARS, Tifton, GA, USA

## Abstract

Many plant ESTs have been sequenced as an alternative to whole genome sequences, including peanut because of the genome size and complexity. The US peanut research community had the historic 2004 Atlanta Genomics Workshop and named the EST project as a main priority. As of August 2011, the peanut research community had deposited 252,832 ESTs in the public NCBI EST database, and this resource has been providing the community valuable tools and core foundations for various genome-scale experiments before the whole genome sequencing project. These EST resources have been used for marker development, gene cloning, microarray gene expression and genetic map construction. Certainly, the peanut EST sequence resources have been shown to have a wide range of applications and accomplished its essential role at the time of need. Then the EST project contributes to the second historic event, the Peanut Genome Project 2010 Inaugural Meeting also held in Atlanta where it was decided to sequence the entire peanut genome. After the completion of peanut whole genome sequencing, ESTs or transcriptome will continue to play an important role to fill in knowledge gaps, to identify particular genes and to explore gene function.

## 1. Introduction

Legumes are a diverse and important family of angiosperms. With more than 650 genera and 18,000 species, legumes are the third largest family of higher plants and are second only to grasses in agriculture [1]. Peanut is one of the major economically important legumes and is widely grown in the southern United States, and countries like China, India, and in South and Central America and Africa. On a global basis, peanut is a major source of protein and vegetable oil for human nutrition. China and India together produce almost two-thirds of the world's peanuts, and the US produces about 6% (http://faostat.fao.org/). Nearly two-thirds of global production is crushed for oil, and the remaining one third is consumed as food. From 2004 to 2006, peanuts were grown on an average of 21.49 million hectares worldwide with production totaling 32.98 million metric tons (http://www.nass.usda.gov/Publications/Ag_Statistics/2008/Chap03.pdf). During the same time, the US peanut averaged 571 thousand hectares with production of 1.9 million metric tons. Peanut production also has a significant role in sustainable agriculture in terms of global food security and nutrition, fuel and energy, sustainable fertilization, and enhanced agricultural productivity as a rotation crop. Peanut offers numerous health benefits, but is also one of the primary food allergens. High production cost is another challenge to peanut farmers as exemplified by chemical control of foliar diseases. Food safety concern due to aflatoxin contamination of peanut is another important issue threatening human health.

Genomic research can provide new tools and resources to enhance crop genetic improvement and production [2]. However, genomic research in peanut is far behind those in other crops, such as maize, soybean, wheat, sorghum, and potato due to the shortage of essential genomic infrastructure, tools, and resources. Although peanut is an economically and nutritionally important crop globally, it was virtually unexplored at the genome level. On March 22-23, 2004, scientists with expert knowledge of critical fields in genetics and plant molecular biology participated in a workshop hosted by the Peanut Foundation/American Peanut Council in Atlanta, GA, USA. These scientists reviewed the status of peanut genomic research, which was documented in the book entitled *Legume Crop Genomics *[3] under the auspices of the US Legume Crop Genome Initiative (LCGI). The Peanut Genome Initiative (PGI) was launched at this workshop. An advisory committee, representing the broad interests of industry and the peanut research community, was selected to guide the growth of the PGI. A *Strategic Plan for the Peanut Genome Initiative 2004–2008 *was developed that outlined research goals, objectives, performance measures, and significant near-term milestones in the advancement of this emerging science (http://www.peanutbioscience.com/). Development of expressed sequence tag (EST) database was on the top of the priorities.

There have been tremendous developments and applications from the EST concept proposed by Adams et al. [4]; however, only limited but encouraging progress has been made in peanut. Among the plant genomes, ESTs are currently the most widely sequenced nucleotides in terms of the number of sequences and the total nucleotide count. ESTs provide a robust sequence resource that can be exploited for gene discovery, genome annotation, and comparative genomics. In this paper, we will summarize the progress that has been made and the applications that have been developed, particularly the chronological events of development of Peanut Genome Initiative (PGI) from EST to whole genome sequencing as archived in http://www.peanutbioscience.com/.

## 2. The Past and Present of Peanut EST Projects

EST was an important research area of functional genomics during the early days when only a few plant genomes were sequenced and before the development of the second generation of high throughput sequencing technology. It was especially important for major crops or economically significant plants with large genomes, such as peanut. EST sequencing was a feasible approach for researcher to gain sequence information for gene cloning, gene expression analysis, and molecular marker development. However, at the dawn of launching the PGI at the historic Atlanta Workshop in March 2004, there was virtually no peanut EST in public databases like NCBI dbEST (GenBank) (http://www.ncbi.nlm.nih.gov/dbEST/dbEST_summary.html/). For example, for plant ESTs in NCBI dbEST (February 15, 2003), wheat (*Titicum aestivum*) had the most with 415,589, barley (*Hordeum vulgare*) was the second with 314,884, soybean (*Glycine max*) had 308,564, maize (*Zea mays*) had 198,034, rice (*Oryza sativa*) had 130,775, *Arabidopsis thaliana* had 178,464, *Sorghum bicolor *had 84,712, and potato (*Solanum tuberosum*) had 94,423. After the launching of the PGI, Luo et al. [5] reported the first peanut EST project and released 1,345 ESTs to GenBank EST database in May 2003 (accession numbers CD037499 to CD038843).

Peanut EST projects started very slow. Two years after the PGI was launched (January 20, 2006), peanut had 2,171 ESTs in the GenBank. At the same time, there were 7,596,977 human ESTs. Among plants, maize had the most ESTs with 662,884, and wheat was second with 600,205. *Arabidopsis thaliana* had 421,027, rice had 407,545, soybean had 356,808, and even *Medicago truncatula* had 221,123. Peanut was dramatically low on the list for such an important crop.

It has been a long journey for the peanut research community to reach a comparable number that we have today (March 1, 2012). Including wild diploid peanut, the peanut community has deposited 252,832 ESTs to NCBI EST database. There are 178,490 for *A. hypogaea *(including 745 for subsp. *fastigiata*), 35,291 for *A. duranensis*, 32,787 for *A. ipaensis*, and 6,264 for *A. stenosperma*. However, there are a total of 72,098,708 ESTs in the public database, and 8,315,295 are human ESTs. Maize has 2,019,114, soybean has 1,461,624, rice has 1,252,989, wheat has 1,073,877, barley has 501,838, upland cotton (*Gossypium hirsutum*) has 297,239, and potato has 249,761. Before the completion of peanut whole genome sequencing, sequencing large numbers of ESTs can create a formidable resource for studies in both biodiversity and gene discovery. Sequence analysis tools have extended the scope of EST analysis into the fields of proteomics, marker development, and genome annotation. Although EST collections certainly are not intended to substitute for a whole genome sequences, the EST resource forms the core foundations for various genomewide experiments, particularly for microarray gene expression study, marker development, and genetic map construction, which could also be useful to assist research to assemble the whole genome.

Peanut ESTs were derived from different tissues, different growth stages and under different abiotic and biotic stresses [5–12]. For instance, Luo et al. [5] construct two cDNA libraries from leaves of the peanut cultivar Tifrunner (C34-24) [13] and A13. This was the first report of ESTs in cultivated peanut. Proite et al. [6] obtained 6,264 high-quality ESTs from four cDNA library leaves and roots of *A. stenosperma* and developed 188 SSR markers. Alves et al. [14] selected ten EST sequences with homologies of interest and obtained 8 SNP markers based on the single-base primer extension method. Recently, Koilkonda et al. [15] developed 3,187 EST-SSR markers, which were obtained from 10,102 potential nonredundant EST sequences, including 3,445 contigs and 6,657 singletons, generated from cDNA libraries of the gynophores, roots, leaves, and seedlings of cultivated peanut.

 Yan et al. [16] constructed a cDNA library of mid-maturation stage cotyledons of peanut cultivar Shanyou 523 and obtained 414 EST sequences. Seventeen percent (17%) of these ESTs-encoded seed storage proteins, including 5 arachin, 2 conarachin, and 6 conglutin-like proteins encoding sequences [17]. Four new members of arachin family and two new members of conglutin family were discovered in peanut.

A full-length cDNA library from immature peanut seed was reported by Wang et al. [18]; over 17,000 ESTs were sequenced, and 10,000 ESTs were deposited to the NCBI EST database [10]. From these sequences, large numbers were storage protein-encoding genes, genes encoding transcription factors, and stress- or pathogen-related proteins. Proteins involved in protein destination and storage represented the largest group and accounted for 28.8% of the total proteins. Genes involved in metabolism accounted for 12.5% of the total ESTs. A large proportion of ESTs encoded proteins with unknown function (20.1%) or proteins with no hits (13.9%) in the data base providing the opportunity for novel gene discovery [10].

To gain a better understanding of the high oleic acid accumulation in a high oleic acid peanut variety E12, a cDNA library was constructed and obtained 12,501 ESTs. A total of 4,074 unigenes were generated from these ESTs. Proteins encoded by these ESTs are involved in a variety of biological processes including aminoacid and carbohydrate metabolism, energy metabolism, transcription, protein translation, and transportation [19, 20]. These EST sequences provide valuable information for gene cloning, such as genes encoding key enzymes for fatty acid and seed storage protein biosynthesis.

In order to understand bacterial wilt resistance in peanut, cDNA libraries were constructed from normal leaves and leaves challenged with pathogens using the peanut line 06-4104 with high oil content (56%) and resistance to bacterial wilt. The differentially expressed genes between normal and pathogen-challenged plants were identified. From the EST sequence information (over 25,000 ESTs), 5,920 unigenes from the normal leaf library and 7,507 unigenes from the pathogen challenged library were identified [21]. Xiao et al. [22] reported construction of 5 new cDNA libraries using a novel line with high oil content and high resistance to bacterial wilt disease. EST sequencing was carried out using these libraries and a total of 63,207 ESTs, representing 14,547 uni-ESTs, were generated. Recently, a total of 63,234 ESTs were deposited to the public database (GenBank dbEST) with accession numbers of JK146921 to JK210154.

## 3. Gene Cloning Based on EST Sequences

One important aim for EST sequencing is to clone the full-length sequences of genes with agronomic value. Fatty acid synthesis is one of the most important traits for the oil crop peanut. The first enzyme complex in fatty acid synthesis in plants is ACCase which is composed of 4 subunits, *BCCP*, *BC*, **α*-CT,* and **β*-CT*. With the help of peanut seed EST sequence information combined with homology cloning, the full-length ORF of these four subunits was cloned from cultivated peanut. The multifunctional ACCase, one peptide containing three functional domains, was also identified from peanut [23]. Plant fatty acid biosynthesis is catalyzed by type II fatty acid synthase (FAS) in plastids and mitochondria. Type II FAS complex contains several enzyme and an important protein, the acyl-carrier protein (ACP). Genes encoding ACP, malonyl-CoA: ACP transacylase, *β*-ketoacyl-ACP synthase, *β*-ketoacyl-ACP reductase, *β*-hydroxyacyl-ACP dehydrase and enoyl-ACP reductase were isolated using a similar strategy. One to five members of genes encoding each enzyme were identified [24]. Five different types of ACP genes that showed little sequence similarity were cloned [25]. Oleosin is an important component of plant oil bodies. The expression and accumulation level of a specific oleosin could influence the morphology of oil bodies and seed oil content [26]. There are 284 ESTs that showed high sequence homology with oleosin from other plant species. These ESTs form 6 contigs and represent 6 subfamilies of peanut oleosins. The full-length ORF of these oleosin genes was also cloned (X. Wang, unpublished data).

Several peanut storage proteins are allergens. From the developed ESTs, 8 allergen genes, *Ara* h1 to *Ara* h8 were identified [27]. LEA (late embryogenesis abundant) proteins which play key roles in the protection of seed desiccation accumulate abundantly in seed during late embryo development. Su et al. [28] reported the identification of 19 LEA proteins belonging to 8 different subfamilies. The full-length ORF of many other genes involved in different biological processes, for example, the anthocyanin biosynthesis-related genes were also cloned in the EST project (X. Wang, unpublished data). Based on the EST sequence information several transcription factors including *AhLEC1*, *AhDREB,* and *AhNAC* were cloned and characterized [24, 29, 30]. Promoters of genes with special expression patterns, for example, seed or developmental stage-specific expression, have potential applications in gene engineering research. Cloning of these genes and their promoters is of great interest. Several seed-specific or developmental preferential genes were identified and some of their promoters were characterized ([10]; X. Wang and Han Xia, unpublished data). Based on a pericarp and testa-specific EST, the full-length ORF of a cysteine protease gene was cloned [31].

Genes that may play roles in general disease resistance or in a specific disease are a great interest to researchers. The plant defensin is a key member of the plant immune system, which could play roles in protecting plants from pathogen attack. Based on the EST sequences of defensin together with RACE amplification of the full-length ORF of peanut defensin was cloned [32]. Using the information of an *A. flavus* responsive EST, a full length peanut PR-10 gene was cloned. This protein showed a low level of sequence similarity with the previously reported AhPR-10 and suggested the identification of a novel peanut PR-10 [33]. From the EST sequencing project, Zhao et al. [34] reported that 237 ESTs showed homology with plant lipid transfer protein (LTP) coding genes. These ESTs could form 5 different contigs representing five types of LTP. Plant LTPs were reported to play roles in a variety of biological processes such as development and reproduction, resistance to adverse environment as well as disease resistance.

## 4. Gene Expression Study Using the EST Resources

Microarray analysis is a powerful tool for global gene expression profiling. The first peanut microarray study was carried out by Luo et al. [35] to investigate the differentially expressed genes in peanut in response to *A. parasiticus *infection and drought stress. This microarray was made of cDNA clone spot-array using ESTs from two cDNA libraries [35, 36]. Later, an oligonucleotide microarray containing more EST sequence information was also designed and used for peanut gene profiling [37]. An oligonucleotide microarray containing 15,744 unique probes was created from 49,205 peanut ESTs. A total of 36,766 probes were designed using the server-based eArray platform from Agilent Technologies, an in situ-synthesized microarray platform. A full description of the array is available at the NCBI GEO (gene expression omnibus) database with accession GPL6661. Recently, Guo et al. [38] reported the use of long oligonucleotide sequences in gene expression profiling experiments to identify candidate genes that confer resistance to *Aspergillus *infection due to upexpression in response to fungal infection. The array platform description can be found at the NCBI GEO database (accession GPL13178). Briefly, oligonucleotides ranging from 60 to 70 mer were designed at the J. Craig Venter Institute (JCVI). The total number of oligonucleotides spotted on the microarray was 6,932, the ultra-GAPs glass slides with 3 replications of each oligonucleotide at different locations on the slide.

There is another report on peanut microarray study [10], which made a cDNA microarray using the EST sequences obtained from immature seeds. This microarray was used to analyze differential gene expression among peanut tissues and organs. Gene expression patterns were also analyzed during peanut seed development. Recently, Zhuang et al. [39] made an oligonucleotide gene chip using more than 100,000 unigenes derived from 454 sequences, using this gene chip for peanut genes expression analysis especially for genes that may be involved in *A. flavus* tolerance [39].

## 5. EST-SSR Marker Development

Guo laboratory and collaborator developed a large number of EST-SSR (simple sequence repeat) markers from peanut EST sequences [40, 41]. About 24,000 were analyzed for SSR discovery. A total number of 881 EST-SSRs were identified, and 251 of them could be successfully amplified from peanut. Most of these SSRs exhibited polymorphism in the wildtype peanut; however, there were only a small number of the SSRs showed polymorphism in cultivated peanut [40]. In addition, 740 SSRs were discovered from 20,160 new EST sequences derived from cultivated peanut pod [42], which are currently not deposited in the database. The amplification and polymorphism of some SSRs were tested both in cultivated and wildtype peanut. Using ESTs from immature peanut seed cDNA library, Song et al. [11] identified 841 EST-SSRs. Part of these SSRs were used to analyze the polymorphism among cultivated and wild type peanuts. From 63,207 ESTs derived from 5 cDNA libraries constructed using bacterial wilt resistance peanut line “06-4104”, 2,643 EST-SSR loci were identified [22]. Qin et al. [41] reported the largest collection of SSRs, a total of 4,576 SSR markers from three sources: published SSR markers, newly developed SSR markers from ESTs and from bacterial artificial chromosome (BAC) end-sequences for construction of a genetic linkage map, and QTL analysis of TSWV resistance.

## 6. Transferability and Comparative Genomics

Gene content and order are highly conserved among closely related species as revealed by comparative genetics. Sequence data obtained from several crop plants indicated homology existing between genomes of two or more closely related genera/species. EST-SSRs have the effective transferability across genera/species in many crops [43–47]. EST-SSR markers have a higher transfer rate than SSR markers from genomic sequences due to conservation of transcribed regions among related species [48, 49]. Mace et al. [50] obtained 51 SSR markers from the Leguminosae family by using *in silico* method and also tested 27 diverse *Arachis* accessions, and 18 revealed polymorphisms. Varshney et al. [51] constructed the first SSR-based cultivated peanut map by using partial SSR markers from the diploid AA genome map [52], and legume anchor markers were developed and compared to maps from *Arachis*, *Lotus,* and *Medicago*. Moretzsohn et al. [53] constructed a B-genome linkage map using microsatellite markers developed for other *Arachis* species and showed high transferability (81.7%). This B-genome map was compared to the A-genome map using 51 common markers. Foncéka et al. [54] reported that synteny analysis between A and B genomes revealed an overall good collinearity of the homeologous LGs.

## 7. Construction of Genetic Linkage Map

Development of a genetic linkage map has been a core effort in the international peanut research community, but progress was slow until 2005. A detailed review was presented by Guo et al. [2]. The first application of EST-SSR markers was to construct a linkage map by using an F2 population obtained from a cross between two diploid species with AA genomes (*A. duranensis *and *A. stenosperma*), and this map contained 170 SSR loci (from both genomic SSRs and EST-SSRs) on 11 linkage groups (LGs) covering 1231 cm [52]. Alves et al. [14] located five candidate genes for resistance on this genetic map. An advanced version of this map was developed later by Leal-Bertioli et al. [55], by further saturation with 369 marker loci. A diploid B-genome map was also established from an F2 population of *A. ipaensis* × *A. magna* covering 1294.4 cm and 149 marker loci, of which 25% of the markers were developed from cDNA libraries [53].

There are very few genetic maps available based on cultivated peanuts because of low levels of polymorphism. The first SSR-based genetic linkage map for cultivated peanut was constructed by Varshney et al. [51] with 135 marker loci into 22 LGs, covering 1,270.5 cm. This map was further updated with 191 SSR loci onto 20 LGs with 1,785 cm genome coverage [56]. Foncéka et al. [54] published an SSR-based tetraploid map of the BC1F1 population of “Fleur 11” × (*A. ipaensis* × *A. duranensis*)^4x^ with 298 loci onto 21 LGs. Another SSR-based composite genetic linkage map of cultivated peanut was published, based on three RIL populations constructed from three crosses with one common female parent, contained 22 LGs with 175 SSR markers [57]. Mondal et al. [58] constructed a genetic linkage map, containing 24 LGs with 109 SSR markers, covering 882.9 cm by using a total of 164 recombinant inbred lines derived from rust resistant (VG 9514) and susceptible (TAG 24) cultivated groundnut parents. Since the turning point of the 2004 Atlanta Workshop, more EST-SSRs are available for community use, but marker density is still very low, and more markers are needed, particularly SNP markers. The most recent map by Qin et al. [41] reports an integrated genetic linkage map of cultivated peanut constructed from two RIL (recombinant inbred line) populations, derived from the cross “Tifrunner” × “GT-C20” and the cross “SunOleic 97R” × “NC94022.” “Tifrunner” is a runner market-type peanut (*Arachis hypogaea *L.* subsp. hypogaea var. hypogaea*) cultivar with a high level of resistance to TSWV (tomato spotted wilt virus), and moderate resistance to early (*Cercospora arachidicola*) and late leaf spot (*Cercosporidium personatum*). “GT-C20” is a Spanish-type breeding line (*A. hypogaea* L. subsp. *fastigiata* var. *fastigiata*) and highly susceptible to TSWV and leaf spots but resistant to aflatoxin contamination. “SunOleic 97R” [59] is from a BC_4_F_5_ selection of a cross of “SunOleic 95R” [60] and “Sunrunner” [61] with high oleic fatty acid but susceptible to TSWV. The breeding line “NC94022” has been reported to have a high level of field resistance to TSWV [62], a selection from a cross between N91026E (an early maturity Virginia type) and a tan-seeded component selected from PI 576638, a *hirsuta* type line. A total of 4576 simple sequence repeat (SSR) markers were used for screening polymorphisms. Two CAPS (cleaved amplified polymorphic sequence) markers were also included to differentiate *ahFAD2A *alleles [63] and *ahFAD2B *alleles [64]. A total of 324 markers were anchored on this integrated map covering 1,352.1 cm with 21 linkage groups (LGs). This map defined 7 homoeologous groups, and 17 LGs were aligned to corresponding A-subgenome (A1 to A10) or B-subgenome (B1 to B4, B7, B8, and B9) of diploid progenitors, which could assist the assembly of peanut whole genome sequencing project. The Peanut Genome Consortium (PGC), an extension of the International Peanut Genome Initiative (IPGI) has initiated a project titled “Creating a Better Future through Global Food Security” and decided to sequence these two RIL populations (http://www.peanutbioscience.com/). Further two major QTLs for TSWV resistance were also identified for each RILs, which illustrated the application of this map [41].

## 8. Peanut Genome and Whole Genome Sequencing

Cultivated peanut is an allotetraploid (2*n* = 4*x* = 40) with little polymorphism at the molecular level [65, 66] as indicated by using traditional markers such as RAPD and RFLP [67, 68]. Pairing in *A. hypogaea* is generally bivalent, with occasional higher-order associations found in crosses among different market types [69]. Cultivated peanut is considered to have originated from a single recent polyploidization event [70, 71], unlike many other natural polyploidy species for which multiple polyploidization events have been identified. In cultivated peanut, the most likely wild diploid progenitors are *A. duranensis* (the A-genome) and *A. ipaensis* (the B-genome) [70]. The peanut genome (2,800 Mb/1C) is large in comparison to the plant models, *Arabidopsis *(128 Mb), rice (420 Mb), *Medicago* (500 Mb), corn (2,500 Mb), and even soybean (1,100 Mb). *A. thaliana* was estimated to have 27,000 genes; rice is predicted to have 30,000 to 50,000 genes [72, 73]; soybean is about 60,000 genes [74]. While the peanut genome probably has a gene number similar to the small-genome species, the large genome size and the complexity makes it difficult to sequence and assemble the whole genome. Therefore, any large-scale discovery, isolation, and deciphering of gene functions for marker development in peanut had been done by relying on other, less cost intensive methods such as the EST project. The EST project focused solely on the development of genomic tools and resources for the peanut community to ensure continued advance in understanding the biology of this important crop species and continued improvement through crop breeding. Moreover, it also constructed a bridge between the peanut genomic research and research on other important legumes for fluid movement of information on gene location and function from other legume genomes to peanut and vice versa, and facilitated exploitation of the high conservation of gene order for comparative functional genomics.

Next-generation sequencing technology and cost reductions have enabled complete genome sequences for many plants, such as rice (2005), maize (2009), sorghum (2009), soybean (2010), and potato (2011). Because of the genome size, sequencing of the entire peanut genome was not feasible until recently. The Peanut Foundation and the American Peanut Council on behalf of international peanut research community and Peanut Genome Consortium (PGC) discussed pursuing a peanut genome sequencing project and related issues on July 12, 2010, at Clearwater, FL, USA, and made the decision to send a delegation to China to initiate discussions with Chinese peanut collaborators for a possible joint sequencing project along with other international partners. Two members from the executive committee Victor Nwosu, Plant Science Program Manager, Global Chocolate Science & Technology of Mars Chocolate, NA, and Baozhu Guo, plant pathologist, USDA-ARS Crop Protection and Management Research Unit traveled to China from September 2–12, 2010 to visit Chinese peanut genomic research collaborator. X. Zhang, Henan Academy of Agricultural Sciences and Peanut Breeder, was the local host and arranged a meeting with Chairman Fuhe Luo, and meeting with others, including X. Wang of Shandong Academy of Agricultural Sciences and Dr. Da Luo of Sun Yat-Sen University. A proposal was made to collaborate and pull resources together to sequence both the tetraploid and diploid peanut genomes. This trip led to another important Atlanta meeting, Peanut Genome Project Inaugural Meeting (http://www.peanutbioscience.com/peanutgenomeproject.html) on December 8, 2010. The delegation briefed the Executive Committee and the PGI agreed in principle to move forward with this collaborative effort to sequence peanut genomes.

The US PGI Committee discussed pursuing a peanut genome sequencing project and related issues on December 8, 2010, at the Winter Conference, Atlanta, and on January 12, 2011, at the Plant and Animal Genome Conference, San Diego, CA, USA, organized by Dr. Howard Shapiro and Dr. Rich Wilson, and tentatively decided to sequence two peanut cultivars and their 100 recombinant inbred line (RIL) progenies in collaboration with Chinese peanut researchers. Nwosu and Guo made another trip to China from March 18 to 31, 2011 to discuss the technical strategies and cost-share with Chinese collaborators, Zhang Xinyou, Han Suoyi, Ma Wenyue, Wang Xingjun, Huang Jiaquan, Tang Ronghua, and Liang Xuanqiang and Chen Xiaoping (on conference call). The group unanimously agreed to join the peanut genome sequencing projects, and signed a Memorandum of Understanding.

A third trip was made by US delegation to China (September 19 to 30, 2011) to discuss the time for launching the peanut genome sequencing project with Chinese collaborators and China Ministry of Science and Technology. The members are Victor Nwosu, Kim Moore, Howard Valentine, and Baozhu Guo. The sequencing and assembly strategies as proposed by BGI (Beijing Genome Institute), are adopting an integrated strategy WGS plus BAC by BAC sequencing with Hiseq2000 technology, and then resequencing and calling SNPs according to alignment to the reference genome with 200 RILs. The SNPs will be used as markers to construct a genetic map for chromosome-level assembly. Detailed information will be posted on http://www.peanutbioscience.com/ and forthcoming project website and other means.

## 9. Next-Generation Sequencing Technologies and EST Project

In recent years, we are experiencing next-generation sequencing (NGS) technologies which are bringing a revolution in both plant and animal genomics research because of continuous developing in technology resulting in continuous decline in sequencing cost and continuous increase in length of sequence read. A number of NGS technologies such as 454, Illumina, and SOLiD have recently become available. These technologies are capable of generating hundreds of thousands or tens of millions of short DNA sequence reads at a relatively low cost. These NGS technologies are being utilized for de novo sequencing, genome re-sequencing, and whole genome and transcriptome analysis. New generation of sequencers, like the Single-Molecule Real-Time (SMRT) Sequencer, HeliScope Single Molecule Sequencer, and the Personal Genome Machine (PGM) are becoming available that are capable of generating longer sequence reads in a shorter time and at even lower cost for sequencing [75].

Despite the completion of several plant whole genome sequences, much of these genomic data are not fully or well understood. A complete genome annotation requires information and knowledge of the whole genome structure, such as transcription start and stop sites, exon-intron structures, splice variants, and regulatory region sequences. Therefore, EST project will continue to play an important role in postgenome sequencing and will apply NGS technologies in transcriptome sequencing. Sanger-based transcriptome sequencing in the form of ESTs has provided an accurate and effective means for annotating many of the more abundant protein-coding genes [76, 77]. However, the limitations of the Sanger sequencing method restrict the utility of these approaches to the annotation of most abundantly expressed genes. For instance, it has been estimated that most EST studies using Sanger sequencing detect only about 60% of transcripts in the cell and thus do not provide a complete representation of the transcriptome [78]. This information gap can be addressed using the next-generation sequencing technologies. For instance, in peanut, Zhuang et al. [39] generated more than 2 million short sequences using 454 sequencing technology, and Zhao et al. [79] used Higseq2000 producing about 14 million short sequences which could form more than 70 thousand unigenes compared to 1,345 ESTs generated by Sanger sequencing in an earlier study [5, 35, 36].

In genome annotation studies, ESTs are aligned to reference genome sequences, thus revealing the presence of exons, introns, exon junctions, and transcription boundaries for the captured genes. The 454 technology has been used to provide annotation information for the genome of maize [80]. NGS applied in transcriptome sequences has achieved sufficient sequencing depth and provides an adequate representation of the cellular transcriptome. NGS technology for peanut transcriptome analysis has been employed to generate a large number of ESTs and unigenes from different tissue and developmental stages of peanut seeds [39, 79]. The huge numbers of ESTs generated by high throughput sequencing technologies will greatly facilitate peanut functional genomic studies.

## 10. Conclusion

Millions of EST sequences from several hundred plant species have been deposited in publicly available expressed sequence tag (EST) databases. Many of these ESTs have been sequenced as an alternative to complete genome sequencing or as a substrate for cDNA array-based expression analyses. Although EST collections are certainly no substitute for a whole genome scaffold, this resource forms the core foundations for various genome-scale experiments before and even after genome sequencing.

The peanut research community has reached the level of 252,832 ESTs in the public NCBI EST database (March 2012). The EST sequence resources have been shown to have a wide range of applications and novel uses. There are two historic events, March 2004 Atlanta Workshop deciding that EST was the priority of peanut genome research and December 2010 the Peanut Genome Project Inaugural Meeting deliberating to sequence peanut whole genomes. The ESTs will continue to be actively sequenced to fill in knowledge gaps and complement to whole genome sequences and annotation. EST sequencing certainly played a key role during the pre-era of whole genome sequence and will continue providing an excellent resource for novel gene discovery and for confirmation of *in silico* gene predictions from genomic DNA sequences. One of the main challenges of low level of genetic diversity has been partially tackled by developing several thousand EST-SSR markers for peanut. Peanut genomic resources continue to build large EST data sets through NGS technologies and thousands of SNP and SSR markers will be developed. Further, it would be possible to integrate the genetic and physical maps in order to facilitate gene cloning and molecular breeding effectively.
